# Pellagra

**Published:** 1911-10

**Authors:** 


### Pellagra

DR. CHILTON THORINGTON of Montgomery, Ala., in the New Orleans *Medical and Surgical Journal,* September 1911, opposes the maize theory of pellagra. He quotes Marie's geographic classification of the United States and comments that this list would apply almost equally well to yellow fever and malaria and emphasizes the infrequency of pellagra in the great western corn states. (However, it is only fair to consider that a corn-producing is not necessarily a corn-consuming state, to the exclusion of an abundance of other food, and that the corn eaten is likely to be of good quality. Ed.) From a study of three hundred and sixty cases, he shows a rapid increase of deaths in the spring and a marked winter decrease, which do not correspond to the conception of a toxic myelitis due to spoiled corn. He notes the relatively large number of insane pellagrins and implies that many cases develop in institutions. (This fact is rather opposed to both maize and entomophoric theories and tends to bear out the editor's hypothesis that pellagra is not of specific nature but a general manifestation of senility, bad nutrition, intestinal putrefaction, and the like). L. O. Howard questions Sambon's theory of conveyance by the sand fly, *simulium reptans*, as that species is not found in the United States, though twenty-seven other species are. (In this connexion, Mr. E. P. Vanduzee of the Grosvenor Library states that several species are found about Buffalo, on both sides of the Niagara River.)

The habitat of the sand fly does not correspond so closely to the distribution of pellagra as that of the mosquito. Pellagra is frequently accompanied with amebiasis, anchylostomiasis, malaria, and the like, pointing to a parasitic origin. Arsenic and quinine are also of value, if not actually curative in the treatment.

Thorington concludes with the suggestion that criminals be subjected to experiments with Balardini's sporosorum maydis; Ceni's aspergillus fumigatus and flavescens; Besta's penicillium glaucum; Tizzoni's streptobacillus pellagrae; as well as Sambon's simulium reptans and various mosquitoes.

---

Dr. Geo. C. Mizell, of Atlanta, *Journal-Record of Medicine*, August, 1911, has investigated two hundred cases of pellagra, all having eaten cotton seed oil and products. In a control series, without definite symptoms of pellagra but many with suspicious symptoms, 48 per cent. had used these products. Of the 48 per cent, 80 per cent. had begun their use only within three years. He believes, therefore that, in spite of the cheapness and high nutritive properties of the oil cake, meal, and the like, its growing use should be checked pending further investigations.

Sambon, *Gaz. degli Osp. e delle Clin.* May 28, 1911, cites the general Italian opinion that pellagra is due to maize or rice. Chantemesse and Ramond, in 1898, isolated a bacterium and claimed to reproduce the disease by inoculating rabbits. Sambon holds, however, that most cases included in the clinical symptom complex, are due to protozoan hematophages, of which those most liable to suspicion are the phlebotomus papatassi and the dilophus febrilis.

